# Chemistry, Physiology and Pathology of Nitrous Oxide—Practical Points, with Comments

**Published:** 1891-05

**Authors:** S. J. Hayes


ARTICLE IV.

### CHEMISTRY, PHYSIOLOGY AND PATHOLOGY OF NITROUS OXIDE—PRACTICAL POINTS, WITH COMMENTS.

BY DR. S. J. HAYES.

### GASEOUS INTERCHANGE.

And, first, as to the effects produced by the inhalation upon the gases contained in the lungs and blood, and for which I have used the term "gaseous interchange."

1. That the expired gas tends to become precisely similar in composition to that inspired, so that, *prima facie*, one would be justified in concluding that no very active decomposition of the gas occurs; and this conclusion is further justified by the researches of Hermann and other observers.

2. That the carbonic acid, instead of being increased in quantity, as is the case in aerial expirations, is rapidly and continuously diminished.

3. That the amount of oxygen is diminished and, in fact, almost entirely disappears.

4. That, as might be expected from the fact, the nitrogen does not take any very active part in the physiological functions, even under ordinary circumstances; in gaseous inhalation, it is removed by diffusion alone, and hence very slowly.

5. That the substitution of nitrous oxide for the other gases does not take place at all equally or proportionately, but mainly at the expense of the oxygen, which is rapidly reduced.

Dr. Amory's experiments upon himself and upon dogs have very much the same result, but were carried somewhat further. He found that the relative amount of carbonic acid inhaled during fifty respirations of the gas was only about half of that found in the same number of aerial respirations.

### CONCLUSIONS.

1. That the progressive loss of carbonic acid in the expired gas is not associated with any accumulation in the blood, and must, therefore, be due either to lessened production in the tissues or to defective absorption by the blood.

2. Now, in order that carbonic acid may be produced, free oxygen, or an agent capable of yielding free oxygen, must be present. But we see that free oxygen is displaced by nitrous oxide, and we know that the latter undergoes no decomposition in the blood.

3. Hence, during inhalation, the process of tissue metabolism and the production of free carbonic acid is in obeyance.

### PULSE TRACINGS.

A comparison of the pulse trace taken from an average healthy individual during the height of anæsthesia, with one taken from the same individual immediately prior to inhalation, shows :

1. An actual increase in the number of heart beats.
2. The greater height of the stroke.
3. The greater sharpness of the initial curve and apices.
4. The almost complete absence of the tidal wave.
5. The accentuation of the dicrotic wave, which is at the same time removed further from the apex of the trace.

I think we may gather from the consideration of these tracings alone (1) that the heart's action is accelerated and slightly increased in force; (2) that the blood pressure is lowered; and this latter point has been proved by actual experiments,

### CAUSES OF DEATH.

1. That when death has occurred it has been due either to syncope or asphyxia.

2. Simple faintness may occur either before the inhalation is complete, apparently from fright, or during the stage of recovery, apparently from the shock of the operation.

3. Fatal syncope has, with very few exceptions, been distinctly traceable to the shock, consequent upon commencing or continuing the operation when the anæsthesia was incomplete, and have, as may readily be imagined, occurred in patients more or less out of health.

4. Hence the importance of never permitting any operation in a semi-anæsthetical condition.

5. The occurrence of asphyxia from laryngeal spasm, or from falling together of the epiglottidean folds, as in chloroform, has not been recorded with regard to nitrous oxide, and does not seem likely to give rise to any trouble. Such a condition, if it does occur, is, of course, to be looked for during the height of anæsthesia.

6. Fatal asphyxia has invariably been the result of some mechanical cause, *e. g.*, regurgitation of vomited matter, slipping of tooth or gag into the larynx, and has therefore usually occurred during or immediately after the completion of the operation.

7. Hence the importance of watching the mouth, as well as the face, during the stage of recovery.

SYNCOPE.

*Recognized by*—Sudden dilation of pupil, extreme pallor, heart failure, muscular relaxation, feebleness of breathing.

*Treatment.*—Prone position, draw out the tongue; artificial respiration.

ASPHYXIA.

*Recognized by*—Increasing duskiness, violent efforts at respiration, gradual failure of pulse.

*Treatment.*—Remove foreign bodies (inversion), or draw the tongue *well* forward, press on the chest, wipe out mucous; laryngotomy followed by artificial respiration.—SILK, *Dental Record.*

In the above article from the *Dental Record* it will be observed that the author fails to recognize the true and *only cause* of asphyxia, and appears to be surprised at the effects as noted during the administration of nitrous oxide gas and hence fails to account for the facts as noted. If he will concede the fundamental truth that asphyxia can only be produced by withholding the free oxygen or by robbing the blood of its due oxygenation, and then use the word asphyxia where he uses the word anæsthesia, he will not find it difficult to account for the results as noted. Nitrous oxide gas is a chemical combination of N2 O and not a mixture as is the atmosphere. The oxygen cannot be free until sufficient heat be applied to decompose the two chemical elements. There is not sufficient heat in an animal to decompose the two elements; therefore the nitrous oxide enters the blood as nitrous oxide and is eliminated as such, and consequently not a particle of free oxygen is imparted to oxygenate the blood or to sustain life. This explains his first notions that "the expired gas is precisely similar in composition to that inspired," which he states is further justified by the researches of Hermann and other observers. It also explains his second notation that "the carbonic acid, instead of being increased in quantity, as is the case in ærial expiration is rapidly and continuously diminished." This must *necessarily* be the case, as free oxygen is the supporter of combustion, and in the administration of nitrous oxide gas the free oxygen is crowded out or withheld, and consequently there must be less carbonic acid evolved. Also, 3d, 4th and 5th notions are in perfect harmony with these laws of nature.

In regard to the pulse tracings as noted, the author seems not to recognize the true cause of the variations in the character and number of strokes or beats of the heart. The neural current in the vasso motor nerves extending into the muscles concerned in the functions of the heart control their action, and the mind influences the neural current. Therefore, the actual increase in the number of heart beats as well as their greater height of the stroke, etc., is due to the excited condition of the mind. If the patient be excited from fear, or struggling for the *force of life*—free oxygen—the pulse will be quick and *forcible* until weakened by robbing the blood of its due oxygenation, so that the cerebral glands cannot generate sufficient animo-electricity or nervo-vital fluid to sustain the action of the heart, and then the tidal wave diminishes, and death is the result, when the asphyxia is carried so far and complete that the *affinity* of the blood for *free oxygen*, has so weakened that it fails to take up the oxygen from the atmosphere. When the patient has once crossed that "dead line," then all the powers and efforts of earth cannot resuscitate the patient.—[ED.—*The D. & S. Microcosm.*

---

ARTICLE V.

